# A Real-Time Tool Positioning Sensor for Machine-Tools

**DOI:** 10.3390/s91007622

**Published:** 2009-09-28

**Authors:** Antonio Ramon Jimenez Ruiz, Jorge Guevara Rosas, Fernando Seco Granja, Jose Carlos Prieto Honorato, Jose Juan Esteve Taboada, Vicente Mico Serrano, Teresa Molina Jimenez

**Affiliations:** 1 Instituto de Automática Industrial, Consejo Superior de Investigaciones Científicas, Ctra. Campo Real km 0.2, 28500 Arganda del Rey (Madrid), Spain; E-Mails: jguevara@iai.csic.es (J.G.R.); fseco@iai.csic.es (F.S.G.); cprieto@iai.csic.es (J.C.P.H.); 2 Instituto Tecnológicó de Óptica, Color e Imagen - AIDO. C/ Nicolás Copérnico 7-13, Apdo. 139, 46980 Paterna (Valencia), Spain; E-Mails: jesteve@aido.es (J.J.E.T.); vmico@aido.es (V.M.S.); tmolina@aido.es (T.MJ.)

**Keywords:** tool localization, laser-tracking, machine-tool, oscillation and deformation measurement, interferometry

## Abstract

In machining, natural oscillations, and elastic, gravitational or temperature deformations, are still a problem to guarantee the quality of fabricated parts. In this paper we present an optical measurement system designed to track and localize in 3D a reference retro-reflector close to the machine-tool's drill. The complete system and its components are described in detail. Several tests, some static (including impacts and rotations) and others dynamic (by executing linear and circular trajectories), were performed on two different machine tools. It has been integrated, for the first time, a laser tracking system into the position control loop of a machine-tool. Results indicate that oscillations and deformations close to the tool can be estimated with micrometric resolution and a bandwidth from 0 to more than 100 Hz. Therefore this sensor opens the possibility for on-line compensation of oscillations and deformations.

## 1. Introduction

**Figure 1.**
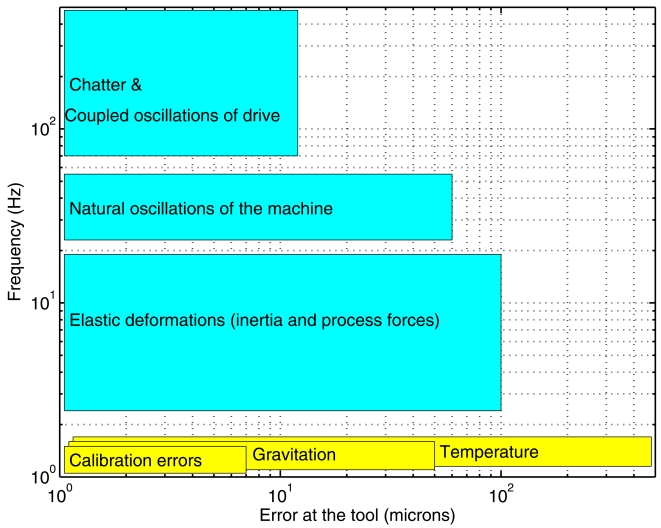
Amplitude versus frequency ranges for different sources of positioning errors in machine tools. Dynamic errors are in cyan, and static or quasi-static errors in yellow.

High-speed machining is a challenging research area with important benefits, such as greater efficiency and lower processing time per machined piece. Compared with traditional machine tools, high-speed machines have increased their speeds and accelerations in around an order of magnitude for both linear and rotational movements. This speed invokes an over-amplification of dynamic errors causing a loss of part finishing quality. Some dynamic errors are the natural oscillations in the machine, and the elastic deformations due to inertia and process forces (see Figure 1). Other inaccuracies come from static errors, for instance, calibration errors caused by inaccurate scale mounting or non-perfect axis orthogonality. Quasi-static errors appear due to temperature gradients and insufficient mechanical stiffness.

Other machine-tools with a totally different construction concept from the traditional ones are those based on Stewart platforms or parallel machines. Although they are based on rigid platforms connected with several prismatic links that can be actuated, these parallel machine-tools are light-weight and can suffer from significant flexion, oscillations and static inaccuracies [1].

The oscillations and deformations induced during machining make very difficult to trace paths accurately. Extensive research has been performed in advanced model-based control algorithms with feedforward and feedback approaches to improve the accuracy of position control loops in machine-tools [2]. Tracing paths with accuracy is specially difficult because the position and velocity control loops are traditionally based on linear scales and motor encoders that are usually located far from the tool's position. The design of a measuring system that could actually estimate in real-time the position of the tool, or at least a point close to it, could represent an important advance for precise machining. From a production point of view, it could improve part quality, rejection rates, and post-finishing stages.

There are several laser-tracking devices that can estimate the position of a moving reference [3–5]. However these systems, mainly devoted to static calibration, alignment and reverse engineering, do not fulfil some specifications demanded by machine tools, such as accuracy, measuring rate, speed and acceleration for tracking. A recent work [6] has proposed the use of a laser-tracking device to guide the trajectory of a robot manipulator realtime so as to improve the absolute positioning accuracy of its end-effector. However, the authors report positioning control errors larger than ±100 microns at the end-effector which are not acceptable in machining applications. Additionally, this approach changes the control law of the manipulator, modifying its standard mode of operation, which can be an important drawback for industrial acceptance.

None of the above presented laser-tracking realizations are able to transfer the estimated position with enough accuracy and in real-time to the numerical control (CNC) of a machine-tool using a standard interface. From the industrial machine-tool industry it would be desirable to have a sensor that directly measures the position of the tool with accuracy, that could be seamlessly integrated and used as any standard positioning sensor (e.g., a linear scale). The sensor should use a standard communication protocol (incremental or absolute), and consequently the machine-tool could maintain the conventional control procedures already developed for machining. In order to achieve this goal we further extended our initial research in laser-tracking technology [7, 8]. In this paper we present the design and test of a new optical measuring system to accurately localize the tool's position and to transfer it in real-time to standard machine-tools.

Section 2, describes the optical system and gives some details about the components and methods to estimate and transfer the position estimation. Section 3, presents some of the tests performed and the results obtained. Finally, we give a discussion and some conclusions.

## 2. System Description

### 2.1. Measurement Principle and Main Components

**Figure 2.**
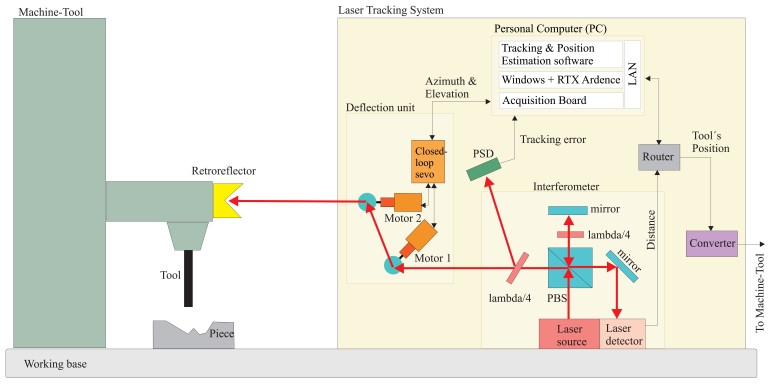
Layout and set-up of the LaserTrack system for tool's position estimation.

Our laser tracking system, named LaserTrack, measures the three-dimensional (3D) position of a reference close to the tool in a machine-tool (see Figure 2 for a conceptual scheme of the LaserTrack system). The reference is a retroreflector-like passive element. An active laser pointing device, positioned on a working base close to the piece and the machine-tool, continuously tracks the reference. The active tracking system estimates the range to the reference, by interferometry, and the azimuth and elevation angles with rotational encoders. A Positioning Sensing Device (PSD) is used to measure the tracking error (offset between the laser spot on the retro and its geometric center). Assuming that a perfect tracking of the reference is achieved, then a polar to cartesian transformation gives an estimation of the reflector's 3D position.

Note that the system estimates the position of the retroreflector, and not the one of the tool itself. However, if the reference is close enough to the tool, then it is expected to obtain good estimations of the actual position, oscillations and deformations on the tool; better than those deduced from the machine-tool's linear scales, at least.

The principal components of our laser tracking system are:

- The *optical reference* to be fixed close to the tool of the machine-tool.
- A laser *interferometer* to measure the distance to the reference reflector.
- A *deflection unit* with two motorized mirrors to bend the laser beam towards the reference.
- A *Position Sensitive Detector* (PSD) for estimating the tracking error.
- A *Personal Computer* (PC) for commanding the mirrors to track the reference and to estimate its position.
- A *converter* for translating in real-time the position estimations into a format that the Machine-Tool can understand.

### 2.2. The Optical Reference

The optical reference used to track the machine-tool position is a retroreflector. It returns back the incoming laser beam through a path that must be coaxial or parallel to the incident one. This reference is a totally *passive* component, i.e., it does not need any cabling for power supply.

Solid-glass and hollow corner cube retro-reflectors were initially used, but their performance was not ideal. Considering we operate at vertex incidence, i.e., the system tries to keep the laser beam pointing on the vertex or center of the retro-reflector, then after a reflection it generates undesirable star-like laser beam cross-sections. In addition, solid-glass reflectors suffered from a secondary reflection on the first facet of the glass. We finally used a cat-eye reflector, which has a smooth spherical reflection surface and therefore provides an unaltered laser beam cross-section which results beneficial for the interferometer measurements.

### 2.3. The Interferometer with Vertex Incidence for Range Estimation

The ranging measurement system is based on the interferometric Michelson-Morley principle. The design and components have been selected to operate with vertex incidence on the retro-reflector. This is necessary to unambiguously determine the position of the retro-reflector. As a consequence the optical arrangement considers a coaxial beam propagation.

**Figure 3.**
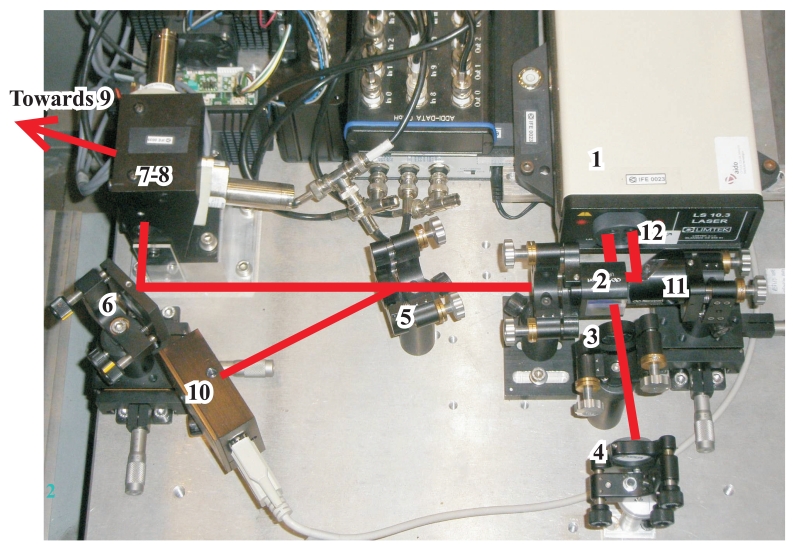
An image of the different optical elements in the measurement system.

Figure 3 shows the implemented optical arrangement. An helium-neon (632.8 nm) laser source (1) with a long coherent length (Limtek LS 10.3) is used as emitter having a 45-degrees polarized beam directed to a polarizing cube (2) which splits the beam in two orthogonally polarized beams: the transmission beam in the reference arm with a vertical state of polarization, and the reflected beam in the measurement arm with a horizontal state of polarization. As it is known, a reflecting element for each arm is necessary in a Michelson-Morley interferometer. Our system uses a first-surface mirror (4) for reference arm and the above mentioned cat-eye retroreflector (9) for the measurement arm [9].

The reference wing (2-3-4) is needed to feed the interferometer detector (12) with a stable reference laser beam. The transmitted beam in (2) reaches mirror (4) after passing through a quarter wave plate (3) whose transmission axis is oriented 45 degrees from the vertical. Mirror (4) returns the beam back towards the polarizing cube (2) passing again through the quarter wave (3) plate. As the quarter wave plate (3) is crossed twice in the reference arm, the polarizing state of the incoming beam in the polarizing cube is rotated 90 degrees and a reflection in (2) is achieved.

The measurement wing (2-5-6-7-8-9) provides the capacity to measure the linear displacement along the beam axis. The reflected beam in (2) is sent to a pair of galvanometric mirrors (7, 8) after a single pass through another quarter wave plate (5) and a 90^°^ bending mirror (6). The galvanometric mirrors (7,8) redirect the beam towards the retro-reflector attached to the machining head (9). After that, the beam travels along the same path in the opposite direction, thus having a new incidence in the quarter wave plate (5). Then, the measurement beam reaches the polarizing cube (2) with a vertical polarization state. Due to the double pass through the quarter wave plate (5), a transmission is achieved on the polarization cube (2).

Both beams, reference and measurement, travel together from the polarizing cube (2) to a final mirror (11) that redirects them to the interferometric detector (12) to evaluate the relative linear displacement of the retro-reflector along the laser beam axis.

### 2.4. The Deflection Unit with Motorized Mirrors

The *deflection unit*, inserted along the measurement arm of the interferometer (7-8 in Figure 3), pursues the steering of the laser beam in order to keep track of the retro-reflector. A good tracking is achieved when the laser beam points to the center of the retro-reflector within an error of ±1 mm. Therefore, for most machine-tool dynamics, it must have a fast response time (*<*1 ms), a high positioning accuracy (below 3 micro-radians) and a sufficient bending range (above ±10°).

We studied several bending technologies for beam deflection. Acousto-optical and electro-optical refractive solutions have very fast response times, but they have a poor resolution (above 100 micro-radians), as well as a limited bending range. The limited bending range (below ±3°) is also the main restriction of mirrors driven by piezoelectric motors. Gimballed mirrors have a good excursion range and accuracy but they have a low angular acceleration. The best performance compromise is probably found with galvanometric motors, which mainly fulfil all the requirements but with a limited angular position accuracy (about 10 micro-radians).

We use in our system two independent light-weight and small size mirrors (15 mm of diameter), each of them commanded by one galvanometric motor (VM2015S from GSI-Lumonics). The cylindrical body of both motors can be seen inserted in the black mounting frame (7-8) in Figure 3. Both mirrors are inside this mounting frame. The first reflecting mirror (7) changes the azimuth of the laser beam and the second mirror (8) controls its elevation.

Each motor is controlled with an stand-alone closed-loop servo (Mini-SAX controller from GSI Lumonics), which was tuned for accurate positioning. This controller has a built-in heating module to keep the motors at a constant temperature. An analog ±3 volt reference is used to command the absolute position of each motor; this signal is generated from the D/A card (14 bits) at the PC. The actual orientation of each mirror is available throughout an analog signal outputted by an absolute capacitive encoder, which is read by the A/D card (16 bits) at the PC. The acquisition board used is the model APCI-3120fromAddi-Data.

We modelled the galvanometric motors for a better knowledge of their actual deflection when a voltage, *V*, is applied to them. A second-order transfer function is fitted to several amplitude step responses. The discrete state-space representation for a sampling interval, Δ*T*, of 200 microseconds is:

(1) $$\left[\begin{array}{r} {\hat{X}}_{1}\\ {\hat{X}}_{2} \end{array}\right]=\left[\begin{array}{rr} 0.9836 & -0.6024\\ 0.5 & 0.23 \end{array}\right]\;\left[\begin{array}{r} X_{1}\\ X_{2} \end{array}\right]\;+\left[\begin{array}{r} 0.5\\ 0 \end{array}\right]V$$

(2) $$\alpha_{\text{motor}}=\left[\begin{array}{r} \begin{array}{rr} 0.37 & 0.49 \end{array} \end{array}\right]\;\left[\begin{array}{r} X_{1}\\ X_{2} \end{array}\right]$$

We used this model to filter out the original angular readings, as provided from the capacitive encoder, which resulted quite noisy (3*σ* = 52 micro-radians). After this refinement, the noise was reduced to less than 1 micro-radian (3*σ*), without adding any time delay.

### 2.5. The Position Sensitive Detector (PSD)

A *Position Sensitive Detector* measures the location of a light spot in one or two-dimensions. We use a two-dimensional PSD to check whether the laser beam is pointing to the center of the retro-reflector. Any deviation of the laser from retro-reflector's center is known as the *tracking error* (*e*).

In our implementation (see Figure 3), we use a bidimensional PSD (10) from On-TRACK Inc (model PSM2-10). It is a 3-layered P-I-N-doped photodiode with a uniform resistivity and four anodes in the first layer. The incident light creates charges on this layer that flow to the anodes. Current intensities at each anode are used to estimate the spot's position in two dimensions. This PSD has a 10 by 10 mm active area and a spectral response from 400 to 1,100 nanometers. The response time is 0.4 microseconds, it has a sub-micron resolution, but a limited all-range accuracy of ±30 microns. We tested the device in our lab and we found that the accuracy was about ±3microns if the laser spot does not separate more than 100 microns from its center.

Inserted in the measurement arm of the interferometric system, our PSD (10 in Figure 3) collects part of the returned beam after a reflection on the air-glass interface of the quarter wave plate (5). During the initial calibration stage, the quarter wave plate (5) is oriented using a tilt-positioning mount, in order to achieve a perfect incidence at the center of the PSD sensitive area (10) meanwhile the beam hits the retro-reflector (9) at its vertex.

### 2.6. Retro-reflector Tracking

**Figure 4.**
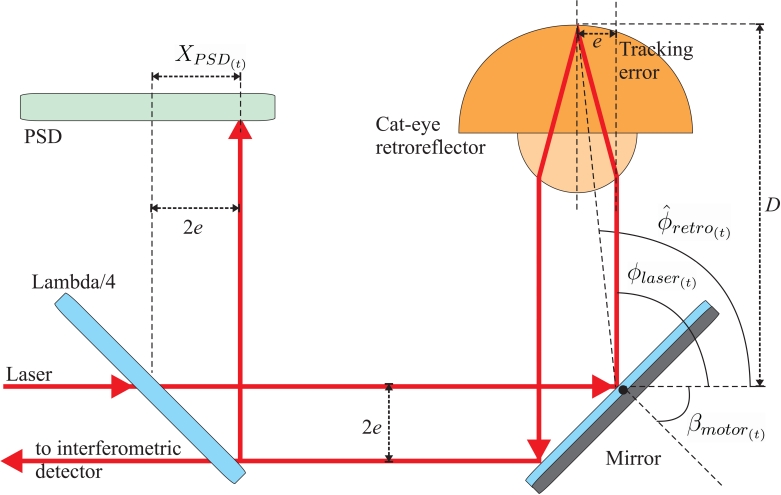
Tracking error representation at retro-reflector and PSD.

As already mentioned, the *tracking error* (*e*) is the lateral deviation of the incident laser with respect to the center of the retro-reflector. If a tracking error occurs, then the incident and return laser beams separate each other by 2 times *e.* This inter-beam separation is the one sensed by the PSD (see Figure 4). We admit a maximum beam separation of about 2 mm, i.e., a maximum *tracking error* of about 1 mm. Larger beam separations can cause the loss of the retro-reflector, because of insufficient overlapping between the reference and measuring beams at the interferometric detector (12 in Figure 3), or due to a blockage of the returned laser beam by some of the mounting frames.

Tracking errors of a millimeter does not mean a poor retro-reflector positioning accuracy. There is a correspondence between the X-Y offset in the PSD (*X_PSD_*_(_*_t_*_)_*, Y_PSD_*_(_*_t_*_)_) and the azimuth and elevation tracking errors, at a given time *t.* So, combining laser beam's azimuth and elevation angles (*ϕ_laser_*_(_*_t_*_)_, *θ_laser_*_(_*_t_*_)_) with the PSD readings and the distance to the retro-reflector, *D*, we can estimate the angular position of the center of the retro-reflector (*ϕ_retro_*_(_*_t_*_)_, *ϕ_retro_*_(_*_t_*_)_):

(3) $$\begin{array}{r} {\hat{\theta }}_{{\text{retro}}_{\left(t\right)}}=\theta_{{\text{laser}}_{\left(t\right)}}+\arctan\left(\frac{Y_{PSD_{\left(t\right)}}}{2.D}\right)\\ {\hat{\phi }}_{{\text{retro}}_{\left(t\right)}}=\phi_{{\text{laser}}_{\left(t\right)}}+\arctan\left(\frac{X_{PSD_{\left(t\right)}}}{2.D}\right) \end{array}$$

Even though the retro-reflector position can be estimated from the PSD and the laser angles, it is preferred to keep the PSD offset as low as possible in other to operate in the most accurate range of the PSD (*+*100 microns). This can be achieved by a tight track of the optical reference.

The tracking error can be minimized by steering the laser beam towards the center of the retro-reflector. As the retro-reflector is expected to move along a predictable trajectory, it is possible to anticipate the laser movement towards the position where the retro is expected to be at next time interval. Some predictive control algorithms for tracking, use the information gathered in its present and previous states to estimate the future velocity and acceleration in Cartesian coordinates (X,Y,Z) [3, 10]. Shirinzadeh proposes a controller that is based on Taylor series to calculate the future position [10]. Whereas, Vinczce minimizes a tracking-error cost function to estimate the future target position [3]. Both algorithms present the disadvantage of being highly sensitive to noise, so a Kalman filters has to be used [11, 12].

**Figure 5.**
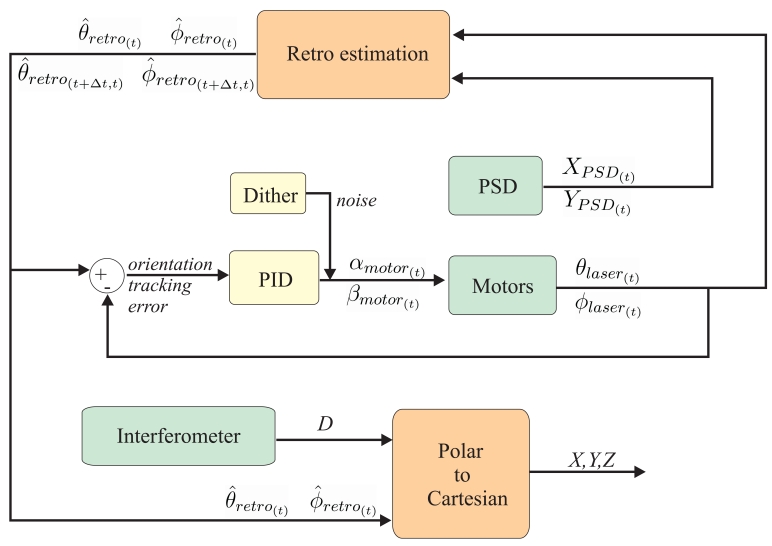
Layout of the tracking and position estimation of retro-reflector.

Our target-tracking algorithm uses the orientation of the laser beam at time *t* (*ϕ_laser_*_(_*_t_*_)_ and *θ_laser_*_(_*_t_*_)_) and the offset readings at the PSD (*X_PSD_*_(_*_t_*_)_ and *YP_SD_*_(_*_t_*_)_) to predict (an interval Δ*T* ahead) the future position in polar coordinates of the retro-reflector (*ϕ̂_retro_*_(_*_t+_*_Δ_*_t;t_*_)_, *θ̂ _retro_*_(_*_t_*_+Δ_*_t;t_*_)_) and its velocity (*ϕ̂_retro_*_(_*_t+_*_Δ_*_t;t_*_)_, *θ̂ _retro_*_(_*_t_*_+Δ_*_t;t_*_)_) based on states and measurements in time t. The discrepancies between the predicted retro-reflector's orientation and the actual orientation of the laser beam is used by a PID controller (*K_p_* = 0.5, *K_i_* = 0.006) to command the motors in order to keep the reference on track. The correspondence between the angles in the motors (*α_laser_*_(_*_t_*_)_, *β_l__aser_*_(_*_t_*_)_) and the laser beam orientation (*α_laser_*_(_*_t_*_)_, *β_l__aser_*_(_*_t_*_)_) is a non-linear function that depends on the actual positioning and orientation of mirrors in the deflection unit. The layout of the tracking loop is illustrated in Figure 5.

Note that a *dither* noisy signal is added to the PID output. This strategy is used to avoid some motor oscillations that could be generated by the limited resolution of the D/A card (14 bits). This angular quantization (47 microradians; 14 microns at 30 cm distance) could cause, otherwise, a continuous oscillation from one angular state to another neighboring state whenever the PID integral error component reaches a given value. The inserted *dither* noise, in amplitude, is half of the actual angular quantization, and it has a probability distribution proportional to the relative closeness to the two neighboring quantization states.

For a proper prediction of retro-reflector estimates with a reduced measurement noise impact, a g-h algorithm [11] is used for estimating the position and velocity of the retro-reflector, assuming a constant velocity model:

(4) $$\text{update equations}\;\{\begin{array}{l} {\hat{\dot{x}}}_{\left(t\right)}={\hat{\dot{x}}}_{\left(t,t-\Delta t\right)}+h\left(x_{\left(t\right)}-{\hat{x}}_{\left(t,t-\Delta t\right)}\right)/\Delta T\\ {\hat{\dot{x}}}_{\left(t\right)}={\hat{\dot{x}}}_{\left(t,t-\Delta t\right)}+g(x_{\left(t\right)}-{\hat{x}}_{\left(t,t-\Delta t\right)}) \end{array}$$

(5) $$\text{prediction equations}\;\{\begin{array}{l} {\hat{\dot{x}}}_{\left(t+\Delta t,t\right)}={\hat{\dot{x}}}_{\left(t\right)}\\ {\hat{x}}_{\left(t+\Delta t,t\right)}={\hat{x}}_{\left(t\right)}+\Delta T{\dot{x}}_{\left(t+\Delta t,t\right)} \end{array}$$

where *x̂*_(_*_t_*_)_ can be the estimated angles in azimuth (*θ̂ _retro_*_(_*_t_*_)_) or the elevation (*ϕ̂ _retro_*_(_*_t_*_)_), and *x̂*_(_*_t_*_)_ their rates of change. The predictions in time (*t* + Δ*T*) based on measurements and states in time (*t*) are *x̂*_(_*_t+_*_Δ_*_t, t_*_)_ and*x̂* (*t*+Δ*t,t*).

The selection of the g-h parameters depends on the dynamics of the target to be tracked, the desired accuracy and the sampling interval Δ*T* [11]. In our implementation, Δ*T* is 200 microseconds, i.e., the tracking control loop is closed at a 5 kHz frequency. The chosen g-h parameters, as a compromise between filtering and response-time, were *g =* 0.062 and *h =* 0.002.

### 2.7. Position Estimation in Cartesian Coordinates

The retro-reflector spatial position (*X,Y,Z*) is calculated by using the distance *D* retrieved from the interferometer, the estimated azimuth and elevation angles of the retro-reflector (now denoted for simplicity as: *θ_retro_* and *ϕ_retro_*), and several parameters that depends on the mirror mounting on the deflection unit:

(6) $$\{\begin{array}{l} X=D\cos(\phi_{\text{retro}})\cos(\theta_{\text{retro}})+p_{x}\\ Y=D\cos(\phi_{\text{retro}})\sin(\theta_{\text{retro}})+p_{y}\\ Z=-D\sin(\phi_{\text{retro}})+p_{z}. \end{array}$$

This calculation (Equation 6) is just a polar to Cartesian coordinate transformation. The Cartesian coordinate system has the origin at the center of the first mirror. This polar-to-Cartesian transformation is offset by a translation*p =* (*p_x_,p_y_,p_z_*) which represents the point where the laser reflects at the second mirror (8 in Figure 3). Point *p* changes when the first mirror reorients for new azimuth angles. It also depends on the physical relative arrangement between both mirrors in the deflection unit. The position and orientation of mirror #2 (8 in Figure 3) with respect to mirror #1 (7 in Figure 3), is defined by a translation matrix, *T*_21_, and a rotation matrix, *R_21_*, which include static terms defined by the mounting dimensions, and dynamic parameters dictated by the orientation of motors (*α_motor_* and *β_motor_*). So, *p*, and consequently *ϕ_laser_* and *θ_laser_*, are non-linear functions of: *α_motor_, β_motor_, T*_21_ and *R_21_*.

A final Kalman filter, assuming a constant velocity model, is used on each of the Cartesian coordinates to minimize some of the measurement errors propagated to this final estimation. A 3 milliseconds delay is introduced by this stage, and a 6.2 microns standard deviation (*σ*) is obtained for a constant retro-reflector position.

### 2.8. Calibration Methodology

As the 3D coordinates generated by the laser system must be coherent with the machine-tool coordinates, we assume that the machine tool has been calibrated for orthogonality, and therefore we calibrate our laser system with respect to the machine-tool coordinates in ideal or static conditions. The calibration is performed in two separate steps:

1. First, we determine the translational and rotational transformation between the machine-tool (*M*) and laser (*L*) coordinate reference systems. The obtained homogeneous matrix *T_M–L_* transforms coordinates in the machine system to the laser one (*M⇒L*). The inverse, $T_{M-L}^{-1}$, does the opposite operation (*L⇒M*).
2. Second, we sequentially move the retro to a regular 3D-mesh of spatial points, and both the machine-tool and the laser retro-reflector coordinates are captured. This mesh is used for a fine correction of new position estimations based on a bilinear interpolation among the 8 closest points in the mesh. This type of calibration eliminates all non-modelled parameters in the laser system such as alignment and mounting errors.

The procedure to obtain the *T_M–L_* matrix consists in positioning the Machine-tool head in three different points [*p*_0_,*p*_1_,*p*_2_]. The first point *p_0_* is arbitrary, the second is just a relative displacement *d* along X axis, i.e., *p*_1_*=*[*p*_0_*_x_+d,p*_0_*_y,_p*_0_*_z_*]. The last point, is just a displacement *d* along Z axis, so *p*2 = [ *p*_0_*_x_,p*_0_*_y_,p*_0_*_z_+d*]. Atypical value used by us for *d* is 20 cm. With the positions of these three points captured from the laser system, we can easily obtain the unitary vectors, along the *M* coordinate axes, seen from the *L* coordinate frame. And finally, we get the homogeneous matrix *T_M–L_* that allows us to translate any (*X, Y, Z*) position from the laser to the Machine-tool coordinate system, and viceversa.

**Figure 6.**
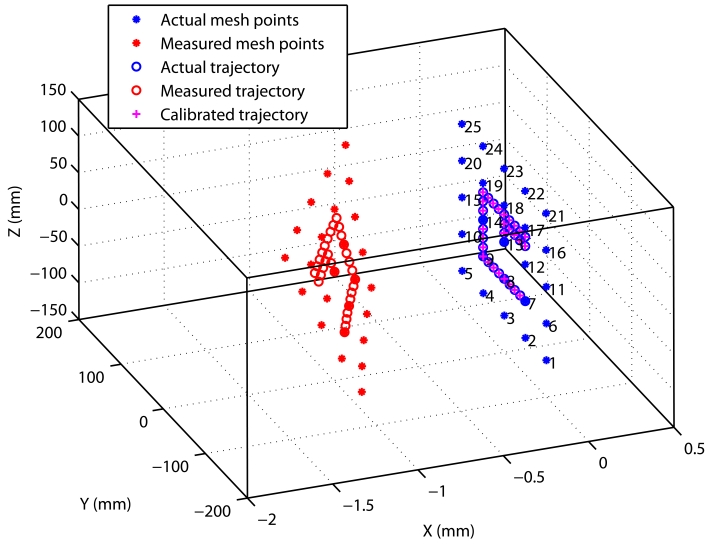
Mesh grid of 5 *×* 5 points used for fine calibrations (asterisks). Test trajectory for checking the calibration performance (blue circles: actual trajectory; red circles: non-calibrated trajectory; magenta pluses: calibrated one).

The mesh of 3D spatial points must cover the expected working volume. The effectiveness of the double bilinear interpolations depends on the separation between points. We are not going to give the details of the implemented bilinear interpolation; however, we will illustrate its performance with a simulation. Figure 6 shows a 5 *×* 5 mesh grid, with points separated by 50 mm. Each of the 25 measured mesh points (red asterisks) corresponds to an ideal position (blue asterisks). Note that there is a significant error of several millimeters. In Figure 6 a simulated trajectory close to the mesh is executed; we show the real trajectory (blue circles), as well as the measured estimations (red circles),and the trajectory points after the bilinear calibration (magenta pluses).

**Figure 7.**
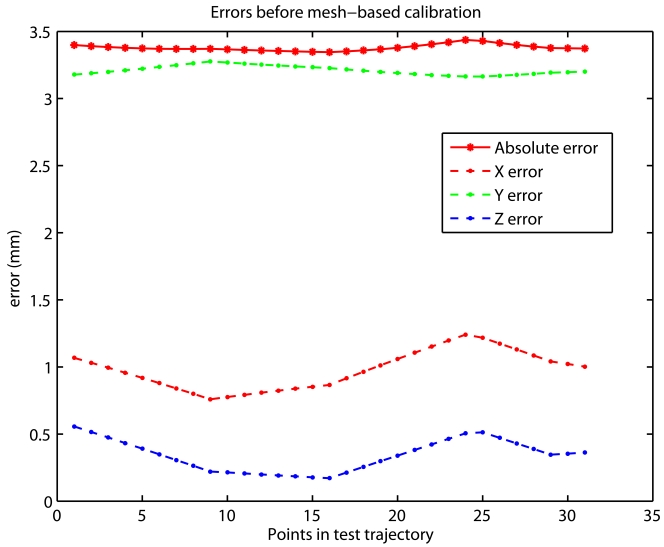

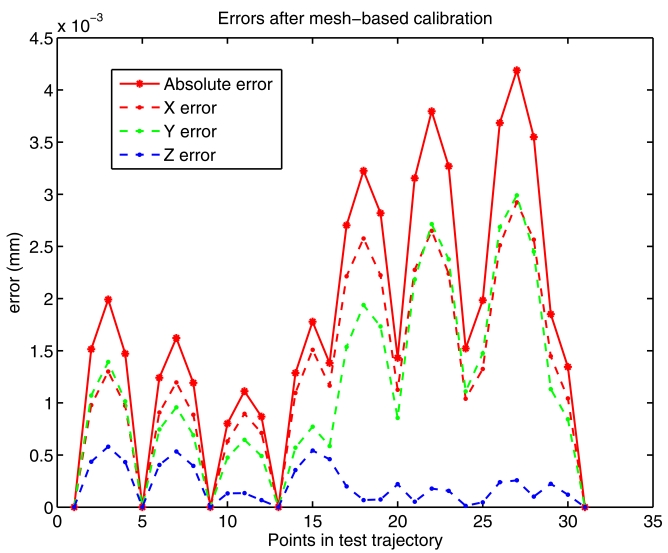
(a) Errors before the calibration in the test trajectory of Figure 6. (b) Errors after the fine bilinear calibration using the mesh points of Figure 6.

Figure 7a,b shows the errors before and after the calibration, respectively. Note that errors after calibration are null when revisiting points in the calibration mesh (points 1, 5, 9, 13 and 31). Between these revisited points, the error grows when the distance from current position to any of the mesh points in the grid increases. From these results, it is clear that the calibration procedure is effective, since a reduction of almost 3 orders of magnitude has been achieved (from 3.5 mm to 4.5 micrometers). These results were obtained with the points in the mesh separated by 50 mm. As stated before if a lower error is required then a shorter mesh-point separation must be used. For example in most of the tests performed in the next section, the mesh grid distance was only 5 mm. This guarantees that the positioning results are not affected by the performance of the calibration stage.

## 3. Tests and Results

Once the methodology of our real-time laser tracking system has been presented, we will describe the set of tests that were performed in order to evaluate the system. First, we will present the performance in lab conditions so as to check the sources of noise and the typical standard deviation of the retro-reflector position estimations. Then, we present how the laser system was installed in two real machine-tools. A large range of tests were performed in these machines, checking the capability of the laser system to detect oscillations or deformations close to the tool. These tests included rotations with different tools, impact tests, and different types of trajectories (straight and circular). Whenever possible, we compared the LaserTrack readings with other sensors such as linear scales and accelerometers.

### 3.1. Quality of Estimations in Static Conditions

**Figure 8.**
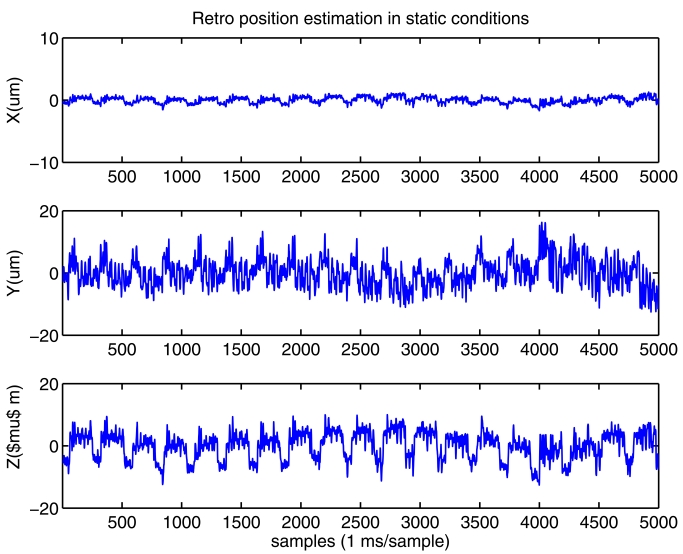

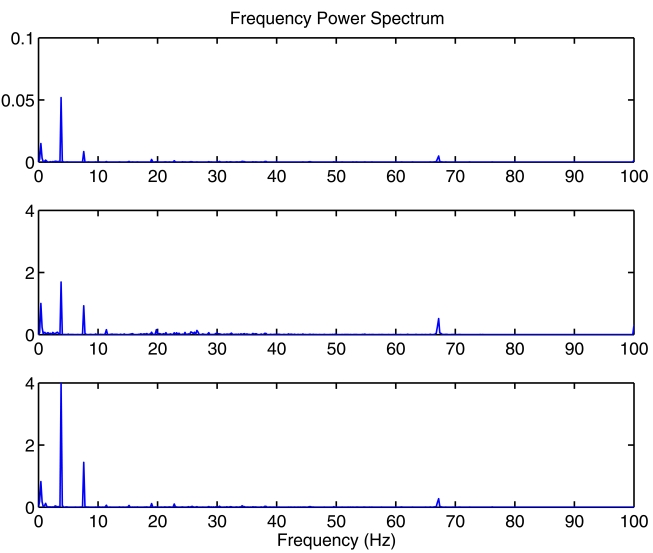
Laboratory tests in static conditions. a) Cartesian coordinate position estimation of a still retro-reflector. b) Its power spectrum.

We know that the most accurate measurement in our polar tracking system is the one along the axis defined by the interferometric laser beam. In all our tests, this axis mainly corresponds, after the polar to cartesian transformation, to axis X. The other cartesian axis (Y and Z) are mainly influenced by the angles measured with the encoders at the galvanometer motors and the tracking error detected by the PSD. So, it is expected a larger error in this angular-dependent estimations. In Figure 8a we show the cartesian position estimations of a static retro-reflector in lab conditions. It is clear that the dispersion in X axis (±1 *μm*) in much lower than in Y-Z axes (±15 *μm*).

The power spectrum of estimations in Figure 8a are shown in Figure 8b. We clearly see, in all axes, frequency components at 4, 8 and 67 Hz. After additional lab tests we found that these oscillations are caused by small but real mirror oscillations that are commanded by the commercially-available close-loop-servo (MiniSAX from GSI-Lumonics) which controls the galvanometric motors. This internal mirror movement exists even inputting a constant voltage reference to the MiniSAX. This mirror motion can not be detected by reading the motor encoders, however it is easily detected by the PSD, and consequently our system interprets it as a retro-reflector movement. This is probably an important drawback of the deflection unit which was selected for our LaserTrack implementation, but it will not seriously affect our oscillation and deformation studies in the machine-tools, which have resulted to have amplitudes as high as 100 *μm*, as we will see along next sections.

Note that frequency components below 4 Hz are a consequence of some laser refractions in air (airflows and temperature gradients). Some high frequency components at 100 Hz are caused by the fluorescent lab lighting that penetrates the 632.8 nm interference filter at the entrance of the PSD. For these reasons, in a final implementation, the laser platform and the path from the deflection unit to the retro-reflector, should be covered and protected in order to reach sub-micrometric accuracy.

### 3.2. Installation of the Laser System in Two Machine-Tools

**Figure 9.**
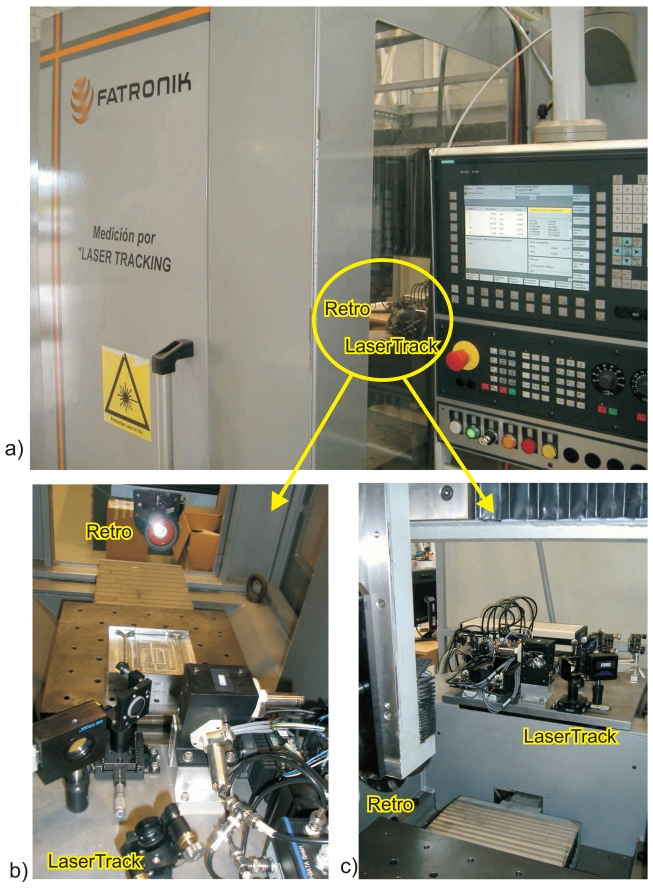
Specially designed machine-tool for Laser Tracking (*Clara-Fatronik*). a) Clara machine-tool with the laser-tracking system installed inside its body. b) and c) are two detailed photos of the retro-reflector setup and the location of the laser-tracking platform.

The LaserTrack system was tested in two Machine-tools: *Clara* and *Axia*. The *Clara* system (Figure 9) is a small and special machine-tool that was designed by Fatronik-Tecnalia to verify the laser tracking concept. It incorporates, inside its body, an empty area of 0.5 m^2^ close to the tool, so as to install the LaserTracking platform. It has three linear degrees of freedom, the X axis is on the workbench, and the ram can be moved in the Y-Z plane. Figure 9b,c shows the detail of the installation of the LaserTrack platform on the internal base of the machine; the retroreflector is attached close to the tool on the ram. Clara uses a Siemens numerical controller with motor encoders for position and velocity control.

**Figure 10.**
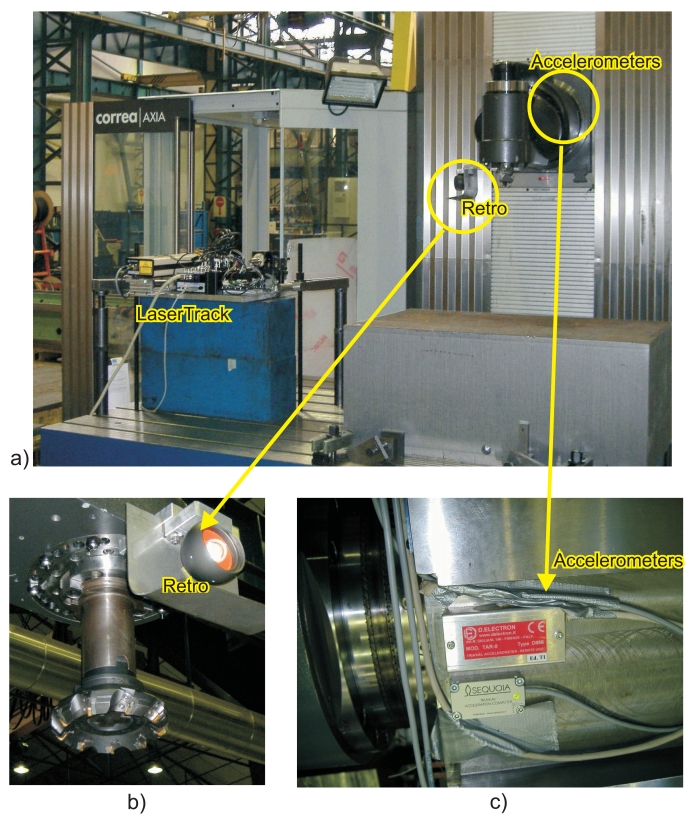
LaserTrack system installed on the AXIA-Correa machine-tool. a) Overall view of the installation. b) The retro reflector is installed very close to the tool. c) Two tri-axial accelerometers are fixed to the ram.

The *Axia* machine-tool is a commercially-available system that is sold by Nicolas Correa. It uses a Siemens numerical controller with linear scales and motor encoders for position and velocity control, respectively. It has 3 linear degrees of freedom and an additional angular axis for tool change and lateral machining. Axis X has a working range of 8 meters, axis Z is above 4 meters and axis Y is limited to 1 meter. We will only use a reduced space of the huge working volume available with this machine. Figure 10a shows the *Axia* machine-tool; a 10 Tn working base (lower blue block) is used to support an additional smaller blue block that holds the laserTrack system. Figure 10b gives a detail of the retro-reflector location close to the tool, and Figure 10c shows two complementary tri-axial accelerometers that were fixed to the ram.

As we wanted the *Axia* machine-tool to be operated in a very open interface mode, we did not use the Siemens numerical control but two innovative controls such as those provided by D.Electron (Italy) and **ISW** (Germany). With these controls we were able to command the machine in the Y-Z plane and to record diverse sensor data, such as linear scales, motor encoders, and accelerometer readings in real-time.

The objective of the tests to be performed in these two machines is not to calibrate or to detect alignment errors in the machines, but mainly to demonstrate that a laser tracking system is able to detect in real-time the oscillations or deformations that the machine-tool by itself cannot detect, and to transmit this information in real-time to the machine for a potential compensation.

### 3.3. Rotations

In order to excite small oscillations in the Axia machine-tool, we make the spindle to rotate at different speeds and with different tools. We tested rotations speeds of 500, 1,000, 2,000 and 4,000 r.p.m. (revolutions per minute), which accounts for 8.33, 16.66, 33.33 and 66.66 Hz. Those rotation speeds are typical in machining; their associated oscillation frequencies, which we want to excite, are close to the natural oscillation frequencies of the machine-tool. We used a drill-tool of 8 mm diameter in order to excite small oscillations, and also a light-weight eccentric tool to generate oscillations of larger amplitude.

We measured the oscillations in parallel with three measurement systems: LaserTrack, the linear scales of the Axia machine, and a couple of tri-axial accelerometers fixed on the ram. One accelerometer belongs to the company D.Electron (Italy) and the other Se-TAC to Sequoia Automation enterprise (Itaty). The sampling time of recorded measurements was 1 millisecond for all sensors.

The amplitude of oscillations with the drill-tool was very limited as expected. The LaserTrack system was able to detect oscillations along its most sensitive measurement axis, X, with an amplitude of only 1–2 micrometers. The linear scales and the accelerometers were not able to detect these small oscillations. The Y and Z axis of LaserTrack were also unable to detect these small oscillations due to the intrinsic measurement noise in these two axes as already commented in Section 3.1.

**Figure 11.**
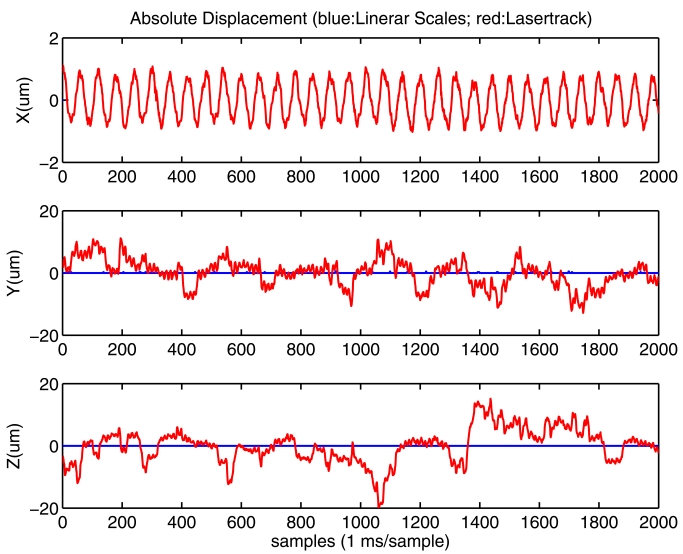

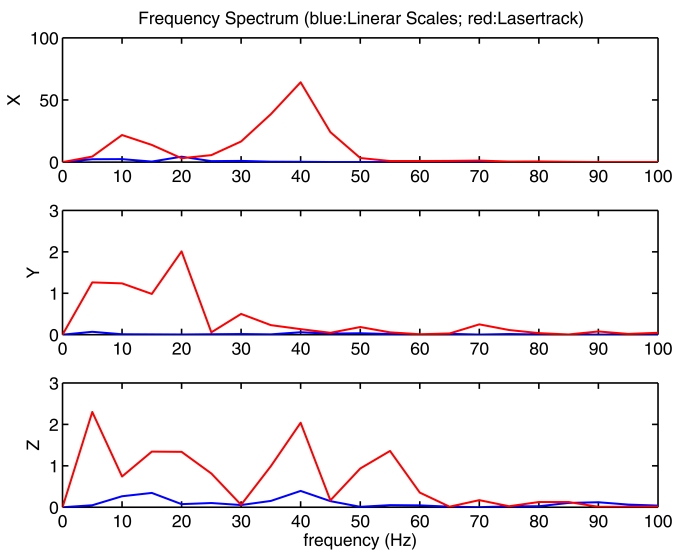

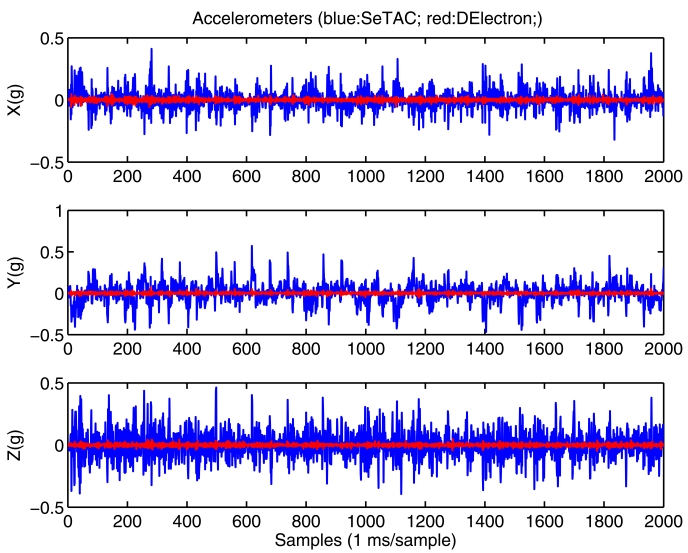

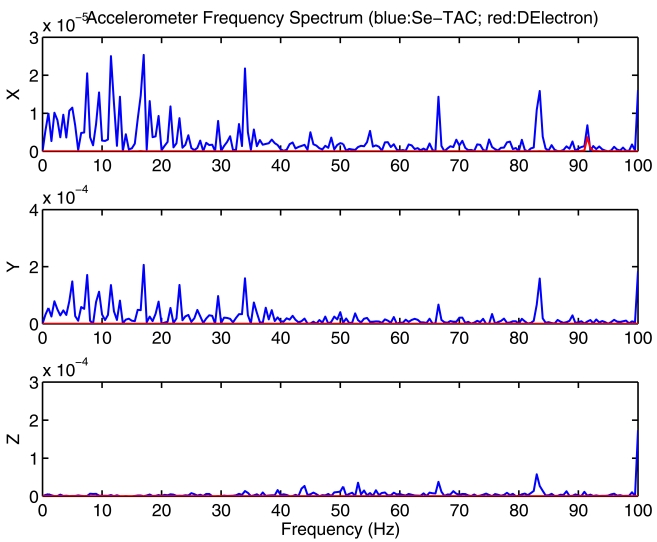
Measurements made with the Axia machine-tool at a constant position with an eccentrical tool rotating at 1,000 r.p.m (16.66 Hz). a) Cartesian positions measured along 2 seconds with LaserTrack and the linear scales. b) Power spectrum of positioning measurements above. c) Accelerations measured by two types of tri-axial accelerometers (DElectron and Se-TAC) fixed to the machine tool ram. d) Power spectrum of acceleration measurements above.

With the eccentric tool the amplitude of oscillations were a little bit larger, up to 8 microns at maximum speed. For speeds above 1,000 r.p.m. some of the accelerometers started to register valid data. In Figure 11 we show the measurements captured for the eccentric-tool rotating at 1,000 r.p.m. We see in Figure 11a that LaserTrack clearly detects an oscillation along X axis of ±1 *μm*. Y and Z only detects miniSAX control noise. The frequency spectrum in Figure 11b reveals a 16 Hz peak, in X axis, corresponding to a rotational speed of 1,000 r.p.m. We can also see in Figure 11c,d that accelerometer readings are very noisy, however the SeTAC accelerometer is able to partially detect the 16 Hz frequencies in X and Y coordinates, as well as other components that are not present according to the LaserTrack X measurements.

**Figure 12.**
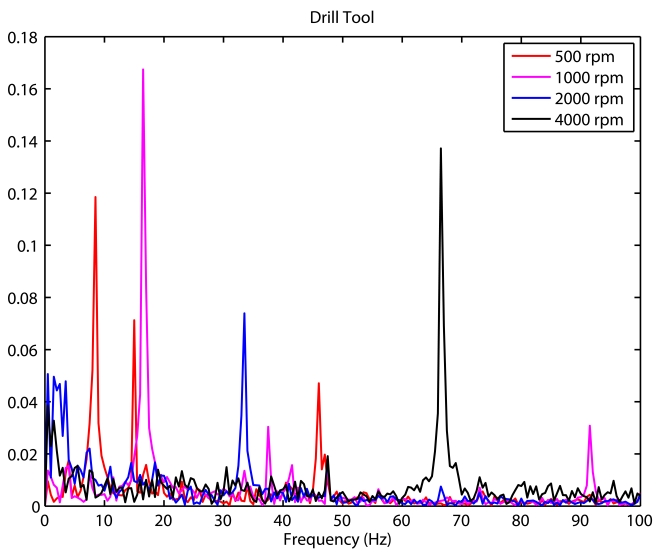

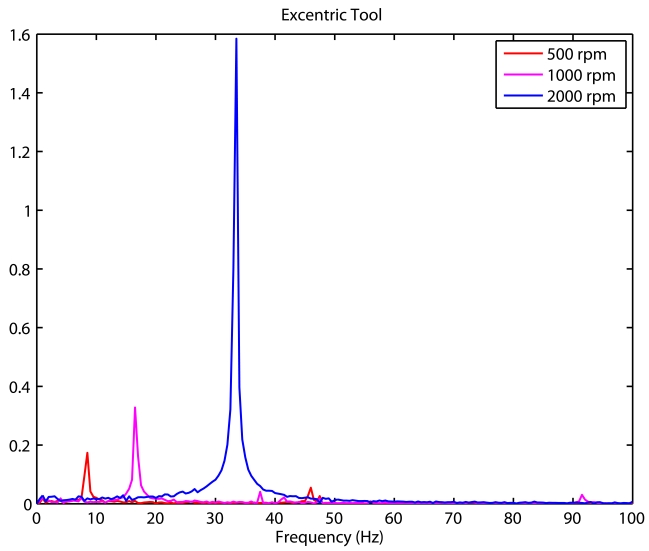
Oscillations measured with LaserTrack along X axis. a) Oscillations caused by a drill-tool rotating at 4 different speeds. b) Oscillations caused by an eccentric-tool.

In Figure 12 we can see the overall rotation results as detected by LaserTrack along X axis. Figure 12a shows the frequency peaks, for the drill-tool case, at four different speeds. Even with oscillation amplitudes below 2 microns, we can see that LaserTrack detects these oscillations. When the eccentric tool is used (Figure 12b), the frequency peaks are much clearly detected, and a larger amplitude is shown at higher rotational speeds.

### 3.4. Impacts

The above-presented rotational tests only excited oscillations with amplitudes below 8 microns. In order to excite larger oscillation amplitudes, the spindle of the machine-tool was hit with an instrumented hammer (model 480C02 from Piezotronics).

**Figure 13.**
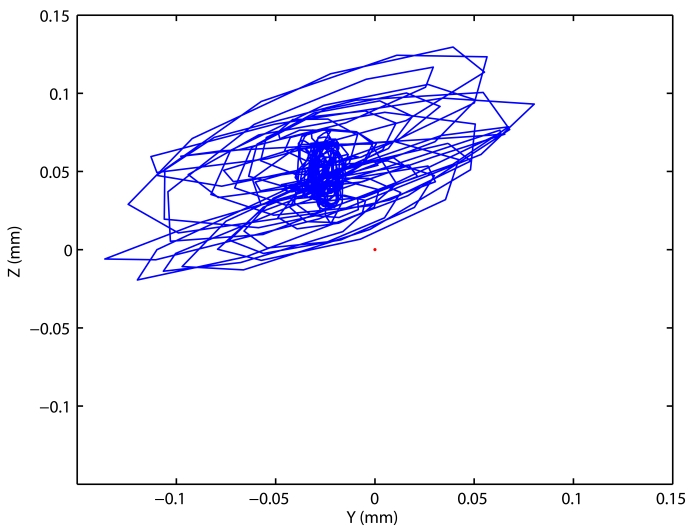

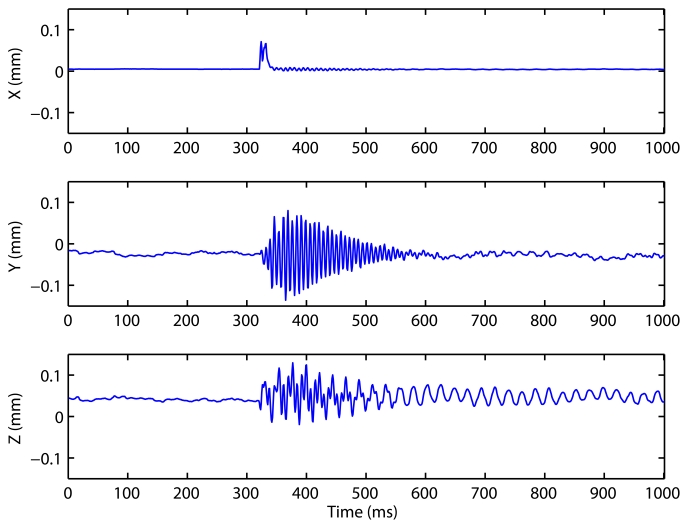

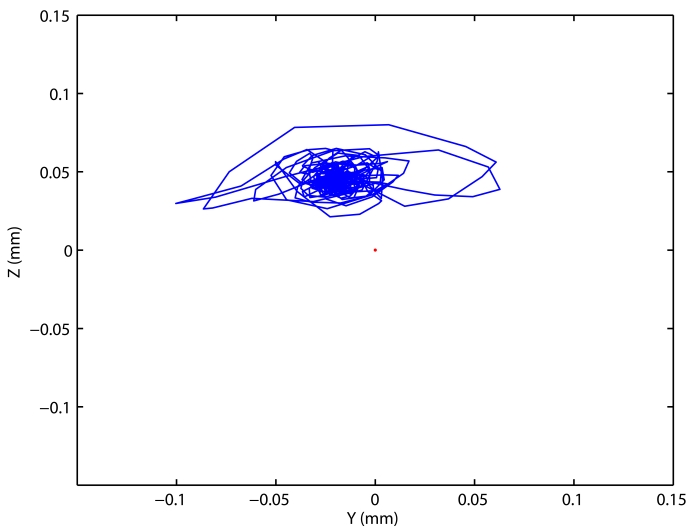

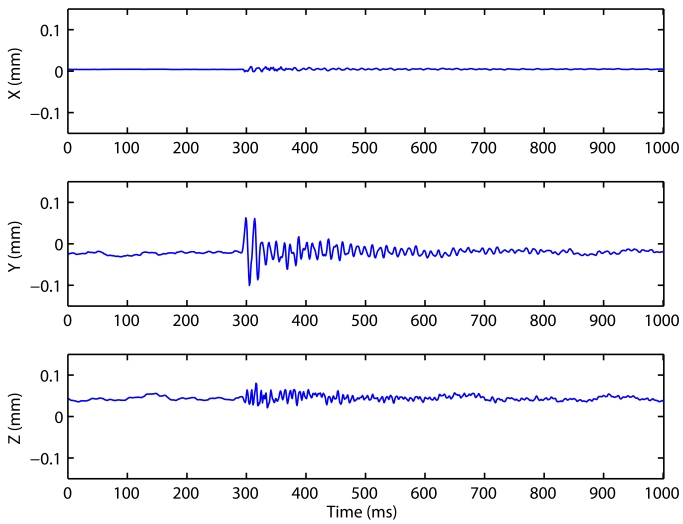

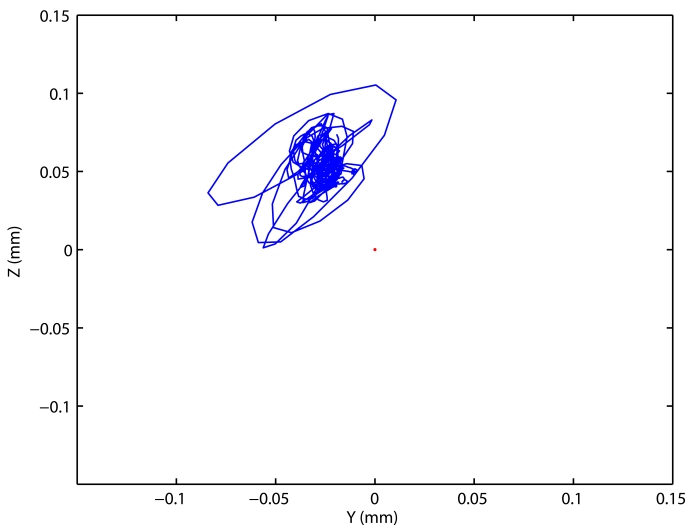

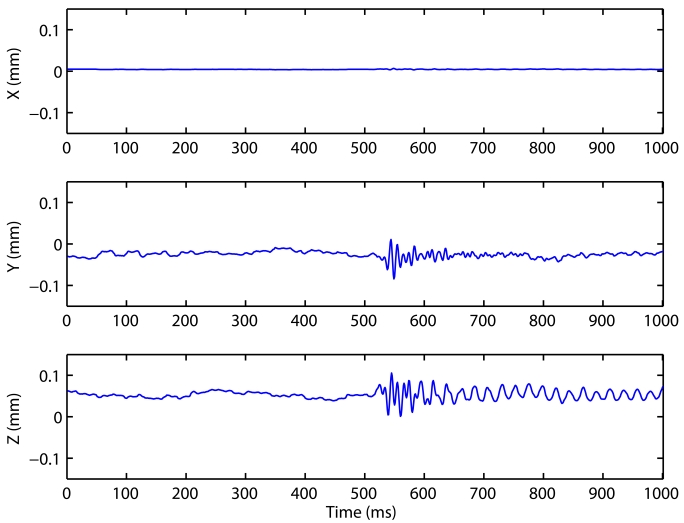
Oscillations measured in Clara machine-tool with LaserTrack, provoked by hammer impacts along three different directions: a) and b) Impact along X axis; c) and d) Impact along Y axis; e) and f) Impact along Z axis.

First, we made the impact tests in the Clara machine-tool. Several impacts along each of the three directions X, Y and Z were recorded with LaserTrack system. In Figure 13 we can see the results of each of the three impacts. The amplitude of oscillations in some axes reaches ±100 *μm*. The oscillations are detected not only with the most sensitive LaserTrack axis, X, but also with axis Y and Z. In Y and Z they have a resonant frequency of 44 Hz; however, X axis has an over-damped behavior.

Other impact tests were done with the Axia machine-tool. In this case we recorded the oscillations with the LaserTrack system, and also with the linear scales of the machine, and the Se-TAC and DElectron accelerometers. The purpose of this parallel registration is two fold: (1) to ratify with the acceleration measurements that the machine head moves as LaserTrack says; and (2) to demonstrate that the linear scales can not correctly see the oscillation in the spindle.

**Figure 14.**
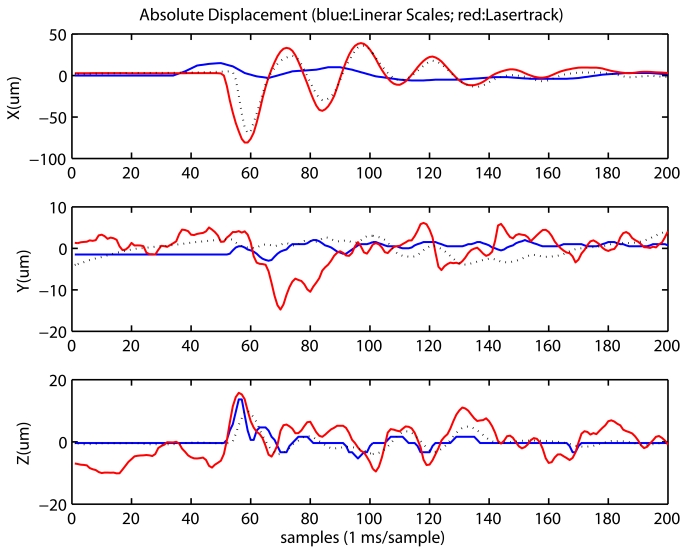

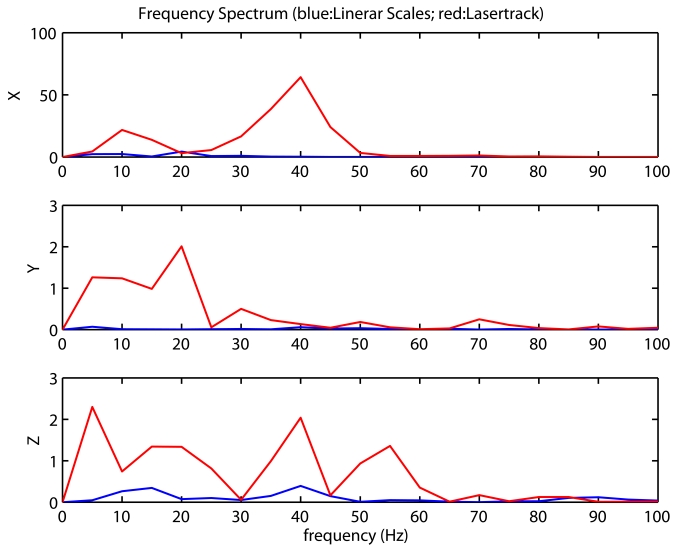

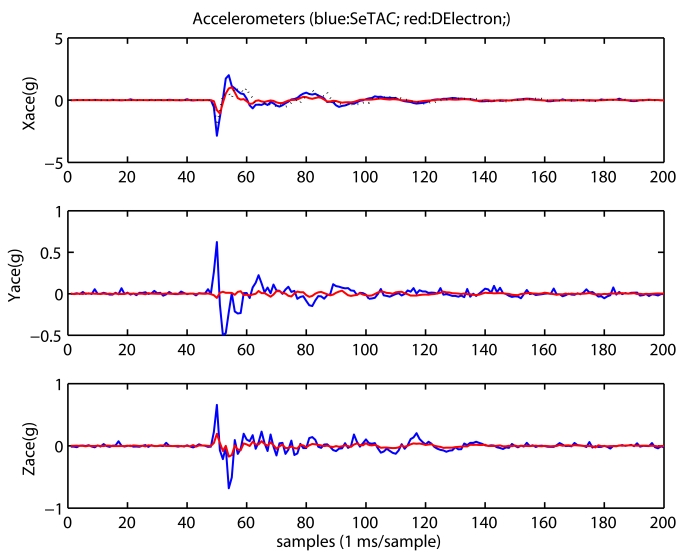

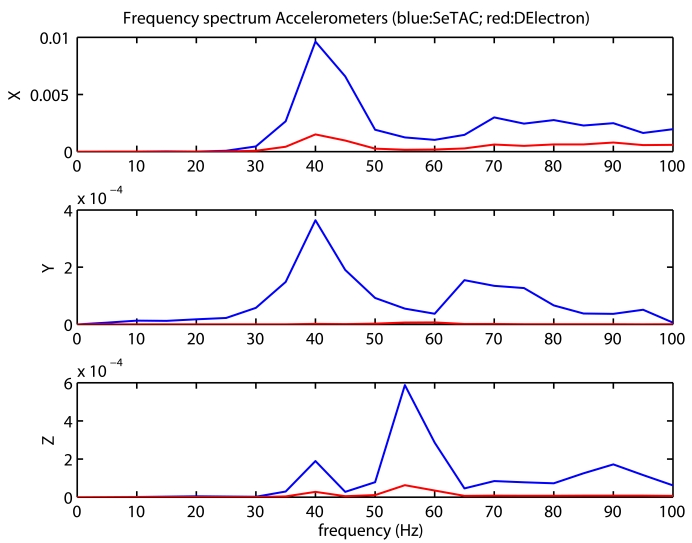
Oscillations measured in Axia machine-tool with LaserTrack, Linear scales and accelerometers. The impact is made along X axis. a) Position registration by LaserTrack and Axia linear scales; with dotted lines it is also included a double integration from accelerometers. b) The power spectrum of above measurements. c) Accelerations registered with Se-TAC and DElectron accelerometers; it is also included, in X axis with a dotted line, the double differences of LaserTrack X measurements. d) The power spectrum of above accelerometer readings.

Figure 14 shows the registered data for an impact along axis X. We see that oscillations above 20 microns only occurs along X axis. In Y and Z there are some minor oscillations with resonant frequencies about 40 and 55 Hz. It is interesting to note that the oscillations in X, as measured by LaserTrack, are confirmed by the Se-TAC accelerometer after a double integration (see dotted line in X plot of Figure 14a). In this case, the oscillation frequency was 40 Hz as seen by both the LaserTrack and both accelerometers. It is remarkable to see how the linear scales, perceive some movement, but does not capture at all the oscillations that are registered by LaserTrack and the accelerometers.

### 3.5. Circular and Straight Trajectories

Two tests were performed under static operation, i.e., keeping the machine-tool at a fixed position. The next tests will involve the movement of the head of the machine-tool along circles and straight lines. We will compare the LaserTrack and Linear scales readings in order to detect flexion or oscillation during the displacement of the ram.

**Figure 15.**
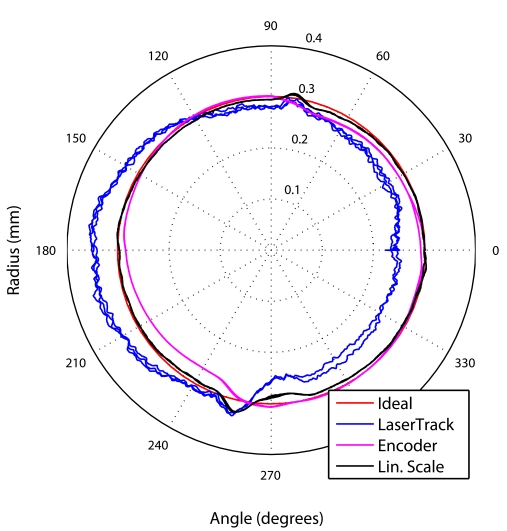

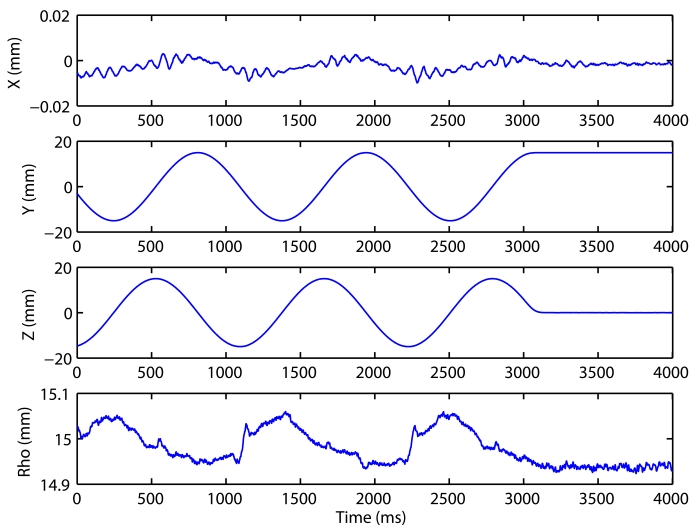

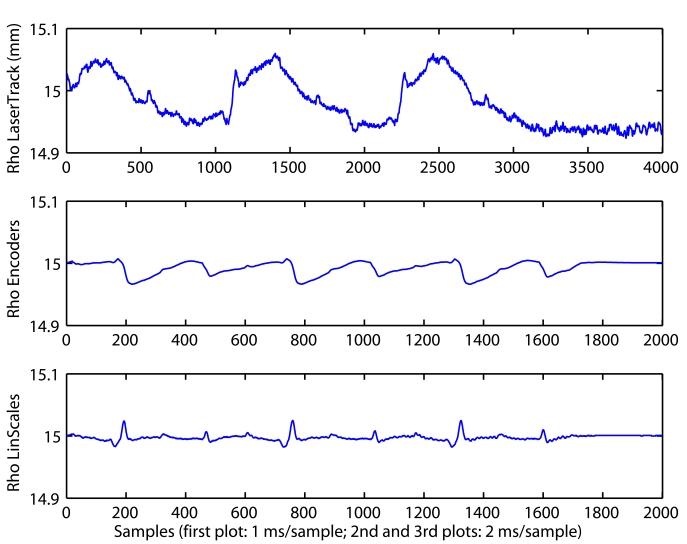
Circular test in the Y-Z plane with the Axia machine-tool. Speed of 5,000 mm per minute (mm/min). a) Polar representation of circular trajectories by three measuring devices: LaserTrack, Encoders and Linear scale. The angular units are in sexagesimal degrees and the radius in millimeters. b) LaserTrack readings in cartesian coordinates versus time. c) Radius estimated by the three measuring systems vs. samples.

Several circular tests were performed at Clara and Axia machine-tools at different speeds (1,000, 5,000 and 10,000 mm per minute). The circles were programmed to have 15 mm of diameter and to be contained in Y-Z plane. Figure 15a shows in a polar plot, the registered circular trajectories after completing 3 iterations. It is shown the ideal circle, the linear scales readings, and the LaserTrack measurements, as well as, the trajectory as estimated from the motor encoders.

Figure 15b shows the information captured by LaserTrack in Cartesian coordinates. There is a small oscillation in X axis with an amplitude of about 10 microns. Below in this figure, we also show the radius (*Rho*) of the circle measured by LaserTrack. The dispersion around the ideal 15 mm value is of ±60 microns, as it can also be observed in Figure 15a. Figure 15c, represents the radius measured by the three measuring methods: LaserTrack, linear scales and motor encoders. The largest deviations corresponds to the LaserTrack system.

As seen in Figure 15a, the Linear scales gives a trajectory that mainly overlaps the ideal circle, except at the north and south of the plot, where some control problems exist. This relative good reference tracking is normal since the position control loop is being executed using the information from the linear scales. Motor encoder readings differ from the ideal circle but this offset is admissible since this nformation is only used to close the velocity loop in the machine. However, the laser tracking system differs significantly, as noted before, from the ideal circle in ±60 microns. We know that LaserTrack estimations in Y and Z have a background noise of about ±15 microns, but even so, it is clear from Figure 15a that LaserTrack is detecting a trajectory that is not perfectly circular. Note that it is not an offset error in Y axis, because the match in the north and south control faults is perfectly synchronized with the linear scale readings. Therefore LaserTrack has demonstrated that the circle executed with this machine-tool is only accurate in a 100 microns window. Similar errors are detected at rotation speeds of 5,000 m/min, but even larger errors (±80 microns) are detected at speeds of 10,000 m/min.

**Figure 16.**
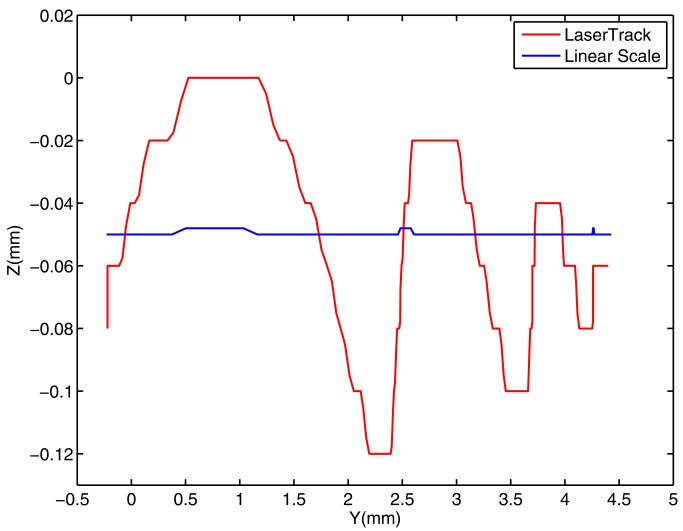

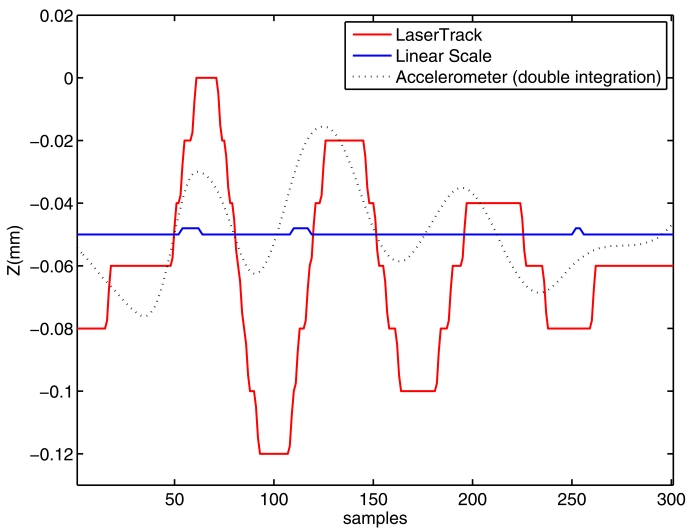

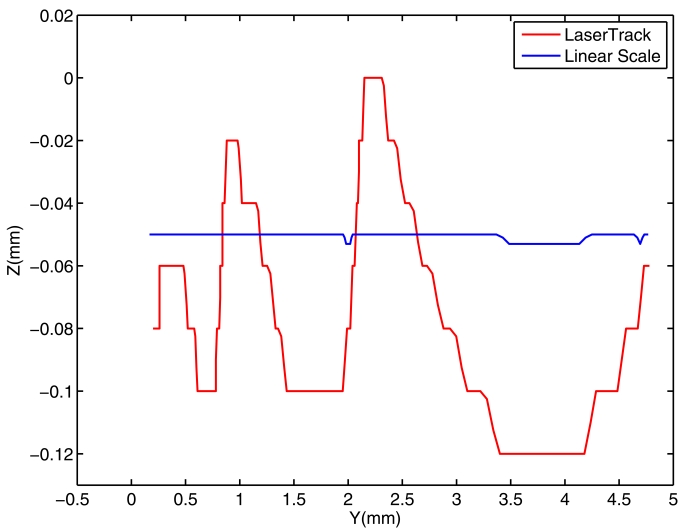

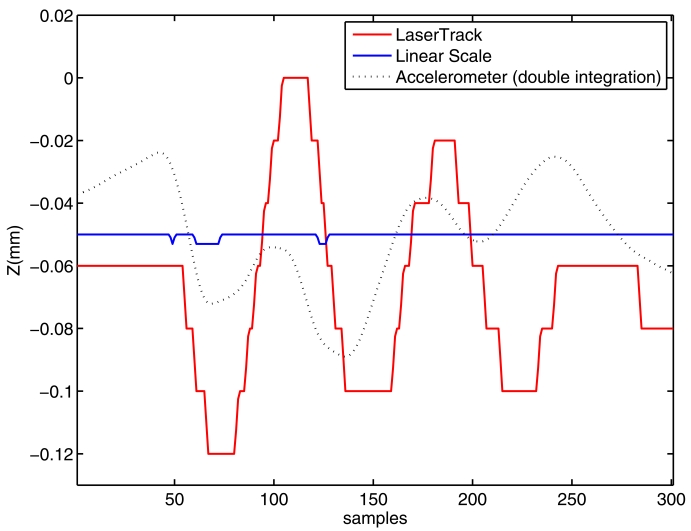
A 5 mm linear trajectory along Y axis. a) A first straight trajectory in Y-Z plane, b) Again, the first trajectory but plotting Z versus time (1 ms/sample) and the result of integrating the Se-TAC accelerometer. c) A second straight trajectory in Y-Z plane, d) The same as in “b)” for the second trajectory.

Additional tests were performed by moving the machine-tool head along straight lines. We tested displacements along Y axis and Z axis, and registered the LaserTrack estimations, the Linear Scale readings, and the accelerations from Se-TAC. In Figure 16 we can see the results for a 5 mm linear displacement along Y axis.

In Figure 16a it can be seen, from the LaserTrack readings, that there is a clear oscillation in Z axis which has an amplitude of 120 microns. Linear scales does not detect almost any movement in the Z direction. However as the accelerometer readings indicate after a double integration (see Figure 16b), this oscillation exists. We remark that, in this case, the three data streams are perfectly synchronized; this is because the three sensors were physically connected to the same ISW control, the one used to command the trajectory.

A second trajectory of identical length but in reverse direction was registered. The results can be seen in Figure 16c,d, which basically are similar to the previous test. Both trajectories took about 0.3 seconds to be completed, and looking at the plots we can detect approximately three oscillation cycles, accounting for an oscillation frequency of about 8-10 Hz. This low frequency causes a low acceleration along Z axis, so the accelerometers are able to detect the oscillation but not with the accuracy of LaserTrack.

However, LaserTrack does not detect oscillations in every tested linear displacement. For example, in the Axia machine-tool the straight line tests along Z axis did not contained any oscillation in the Y direction, neither was detected by LaserTrack nor by the accelerometers.

### 3.6. Closing the Position Loop with Laser System

Apart from checking that LaserTrack can detect oscillations and deformations that the machine-tool's linear scales can not detect, the other main objective was to be able to transmit in real-time the positioning information from LaserTrack to the numerical controller (CNC) of the machine tool. As already depicted in Figure 2, our Laser Tracking system includes a “converter” that transforms in real-time the digital position estimation packets, which are sent every one millisecond by a TCP/IP network, into a format that the CNC can understand. This format is the standard incremental sinusoidal quadrature transmission that emulates an encoder in operation. A separate channel exists for each of the three axes, X, Y and Z, of the machine-tool. The detail of how this converter operates is not going to be detailed now, since it is patent pending.

**Figure 17.**
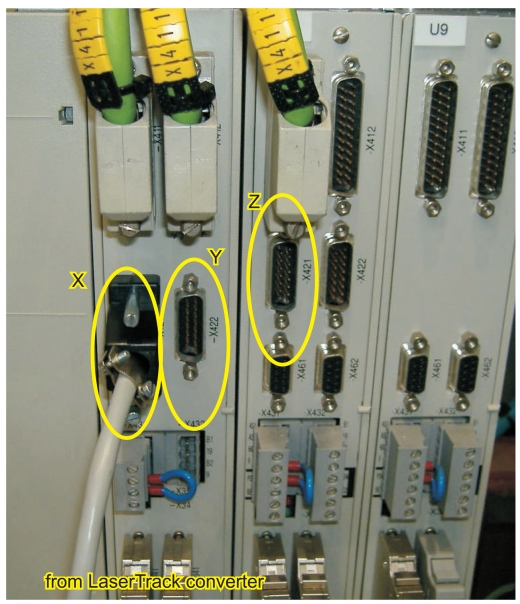
Siemens Simodrive 611 boards in Clara Machine-tool. The X, Y and Z outputs from the LaserTrack encoder converter are connected to the marked connectors in order to close the machine-tool position loop in real-time.

The LaserTrack converter has been successfully tested on both Clara and Axia machine-tools. In Clara, the machine-tool has Siemens motor controllers (Simodrive 611); we connected the LaserTrack output to the X421/422 connectors of the three Simodrive boards (see Figure 17). These connectors are normally used to input the position information coming from the linear scales, but are now substituted by LaserTrack position information. The incremental 3D information that is finally transmitted to the CNC, was compared with the original absolute data available at the LaserTrack PC. We verified that after several minutes of operation performing different trajectories, there were no errors accumulated over time. After this initial check, we configured the machine-tool to close the position control loop with LaserTrack (the linear scales were disconnected) and the motor encoders were used to close the velocity control loop. The machine-tool was programmed to perform several trajectories; the controlled movement was smooth, operating without any problem using our LaserTrack readings. Tests resulted totally successful. As far as we known, this is the first time a real machine tool has been controlled with a laser tracking system.

## 4. Discussion

Although the LaserTrack system has a background noise in Y and Z axes of approximately ± 15 microns, this is mainly a limitation of the quality of the components used in our LaserTrack implementation. Better components could be used in a future prototype. Nevertheless, this drawback did not limit the demonstrated capability of LaserTrack to detect oscillations and deformations. In fact, in our tests, most of the detected oscillatory movements were between 20 and 100 microns. So, all the conclusions found throughout this paper are not limited by the noise in Y and Z axes.

Regarding the implemented converter, which emulates a sinusoidal encoder for real-time position transmission, we would like to remark that it makes our laser tracking system compatible with most of the existing machine-tool controllers. This kind of connection is plug-and-play and it is guaranteed to operate in real-time. No difference at all is detected by the machine-tool controller with respect a real linear scale. The converter could also be used with any other equipment (different to a machine-tool) needing information to be input in real-time throughout one of its decoder inputs.

It could be very interesting to test the system during machining. In this case it is expected to detect even larger positioning errors, flexions and oscillations. Whenever the LaserTrack system needs to be used during the machining of a piece, a telescopic and flexible pipe should be installed from the retro-reflector to the mirror-based deflection device, in order to avoid any laser beam interruption by chips or small particles coming from the milling process. However, this kind of protected operation mode should be further investigated since the accuracy of the system could be deteriorated by the changes of air pressure in the cavity inside the pipe.

## 5. Conclusions

Natural oscillations, elastic, gravitational or temperature deformations, are still a problem to guarantee the quality of machined pieces. In this paper we have presented the design and test of a new optical measuring system to localize and transfer in real-time the tool's position during a machining process. The system tracks and localizes in 3D a reference retro-reflector close to the machine tool's drill. The complete system and its components were described in detail in the first part of the paper.

Several tests, some static, including impacts with a hammer and tool rotations at fixed positions, and others dynamic, by executing linear and circular trajectories, were performed on two different machine tools. The objective of these tests, were not to calibrate or to detect alignment errors in the machines, but mainly to demonstrate that a laser tracking system is able to detect in real-time oscillations or deformations that the machine-tool by itself, using the linear scales, cannot detect. We compared the LaserTrack readings with other sensors such as machine-tool linear scales and two types of tri-axial accelerometers.

Results indicated that oscillations and deformations close to the tool can be accurately detected with LaserTrack (micrometric resolution in X axis, although a ±15 microns dispersion in Y and Z). In general, linear scales are not able to accurately detect oscillation or deformations, and the tested accelerometers are only valid to correctly detect oscillations when having high acceleration values. Low frequency oscillations are not correctly detected by accelerometers. However, the LaserTrack system resulted to have a valid bandwidth starting from 0 Hz to more than 100 Hz.

Another objective pursued was to test the transmission of the position information in real-time to a machine-tool. We used a patent-pending digital to sinusoidal quadrature converter for each of the three cartesian coordinates. Using this device, we believe to have successfully integrated, for the first time, a laser tracking system into the position control loop of a machine-tool. Consequently, the LaserTrack concept opens the possibility for a real-time compensation of micrometric oscillations and deformations. Further research in control strategies must be made in the future in order to analyze the benefits of this mode of operation.
